# How Accurate is Your Sclerostin Measurement? Comparison Between Three Commercially Available Sclerostin ELISA Kits

**DOI:** 10.1007/s00223-015-0105-3

**Published:** 2016-01-09

**Authors:** Isabelle Piec, Christopher Washbourne, Jonathan Tang, Emily Fisher, Julie Greeves, Sarah Jackson, William D. Fraser

**Affiliations:** Bioanalytical Facility, University of East Anglia, Floor 2, Bob Champion Research and Education Building, Norwich Research Park, James Watson Road, Norwich, NR4 7UQ UK; Women Ground Close Combat Review, Directorate of Manning (Army), Blenheim Bld, IDL 27, Monxton Road, Andover, SP11 8HT UK; Norfolk and Norwich University Hospital, Norwich, NR4 7UV UK

**Keywords:** Metabolic bone disease, Sclerostin, ELISA, Clinical utility

## Abstract

Sclerostin, bone formation antagonist is in the spotlight as a potential biomarker for diseases presenting with associated bone disorders such as chronic kidney disease (CDK-MBD). Accurate measurement of sclerostin is therefore important. Several immunoassays are available to measure sclerostin in serum and plasma. We compared the performance of three commercial ELISA kits. We measured sclerostin concentrations in serum and EDTA plasma obtained from healthy young (18–26 years) human subjects using kits from Biomedica, TECO*medical* and from R&D Systems. The circulating sclerostin concentrations were systematically higher when measured with the Biomedica assay (serum: 35.5 ± 1.1 pmol/L; EDTA: 39.4 ± 2.0 pmol/L; mean ± SD) as compared with TECO*medical* (serum: 21.8 ± 0.7 pmol/L; EDTA: 27.2 ± 1.3 pmol/L) and R&D Systems (serum: 7.6 ± 0.3 pmol/L; EDTA: 30.9 ± 1.5 pmol/L). We found a good correlation between the assay for EDTA plasma (*r* > 0.6; *p* < 0.001) while in serum, only measurements obtained using TECO*medical* and R&D Systems assays correlated significantly (*r* = 0.78; *p* < 0.001). There was no correlation between matrices results when using the Biomedica kit (*r* = 0.20). The variability in values generated from Biomedica, R&D Systems and TECO*medical* assays raises questions regarding the accuracy and specificity of the assays. Direct comparison of studies using different kits is not possible and great care should be given to measurement of sclerostin, with traceability of reagents. Standardization with appropriate material is required before different sclerostin assays can be introduced in clinical practice.

## Introduction

Sclerostin is a 190-residue secreted protein member of the DAN/Cerberus protein family. Sclerostin was discovered as a product of the *SOST* gene causing sclerosteosis [1, 2], and van Buchem syndrome [3, 4], and later confirmed in mice in which the *SOST* gene had been deleted [5] or overexpressed [6]. Sclerostin is secreted by osteocytes [7] and articular chondrocytes [8] and its absence favours bone formation by lack of inhibition of the canonical Wnt/β-catenin signalling [9–11], leading to osteoblast differentiation, proliferation and activity [5, 12].

Circulating sclerostin concentrations are altered in metabolic bone diseases. Sclerostin concentrations are increased in disorders such as hypoparathyroidism [13], type II diabetes [14, 15] cancer induced bone disease [16] and Paget’s disease [17] and decreased in primary hyperparathyroidism [18–20] and ankylosing spondylitis [21], although recently increased disease activity in ankylosing spondylitis has been associated with higher sclerostin concentrations [22]. Sclerostin may also play a role of importance in patients with chronic kidney disease associated with mineral and bone disorder (for review see [23]). High concentrations of circulating sclerostin are suggested to be associated with arterial stiffness, cardiovascular calcification and inflammation, leading to higher morbidity and mortality. However, the results so far are controversial.

Measurement of circulating sclerostin may be helpful in the diagnosis of bone remodelling disorders and assessment of therapeutic effectiveness but concordant results between various assays are necessary for clinical trial comparison. Several assays are available for measurement of sclerostin using human blood. We tested and compared three plate-based enzyme-linked immunosorbent assays (ELISA) in serum and ethylene diamine tetra acetic acid (EDTA) plasma samples from healthy young individuals.

## Materials and Methods

### Reagents

ELISA kits were purchased from Biomedica, Vienna, Austria (Sclerostin #BI-20492 lot Y143), R&D Systems, Abingdon, United Kingdom (Quantikine^®^ Human SOST immunoassay #DSST00, lot 318592) and TECO*medical*, Sissach, Switzerland (Human Sclerostin EIA, High Sensitivity #TE1023HS, lot 012455).

### Samples

Anonymised samples from healthy volunteers (aged 18–26 years) were provided by the Ministry of Defence collected in accordance with the Ministry of Defence Research Ethics Committee (MODREC-165). Forty-six serum samples and 27 matching EDTA plasma samples were analysed and sclerostin concentrations were measured following each manufacturer’s instructions.

### Methods and Statistical Analysis

For the Biomedica sclerostin ELISA, 150 μL assay buffer, 20 μL standards, controls and samples and 50 μL antisclerostin antibody were loaded per well. Plates were incubated for 24 h at room temperature (RT = 22 °C) in the dark. The following day, wells were washed five times with 300 µL of the wash buffer provided and 200 μL conjugate was added and incubated in the dark for 1 h. Wells were washed five times with 300 µL of wash buffer, 200 μL 3,3′,5,5′-tetramethylbenzidine (TMB) was added per well, and colour was allowed to develop for 30 min. Stop solution (50 µL) was added and absorbance read at 450 nm with reference at 630 nm.

With the TECO*medical* high sensitivity kit, plates were washed for 2 min at RT with 400 µL wash buffer (provided) and blot dried. Wells were then loaded with 25 µL standards, controls and samples, followed by 50 μL matrix and 50 μL antibody solutions. Plates were sealed and incubated on a shaker at 500 rpm for 4 h. Wells were washed four times with 400 µL wash buffer and then developed in the dark with 100 μL TMB solution at RT for 30 min. The reaction was stopped with 100 μL of stop solution. Absorbance was measured at 450 nm with reference at 630 nm.

For the R&D Systems Sclerostin Quantikine ELISA, 100 µL of assay diluent was added to each well, followed by 50 µL of standards, controls and samples. Plates were sealed and incubated for 2 h at RT on a shaker at 500 rpm. Plates were then washed four times with 400 µL of wash buffer, 200 µL of TMB solution added to the wells and colour was allowed to develop for 30 min in the dark at RT. Finally, 50 µL of stop solution was added to each well and absorbance read at 450 nm with reference at 560 nm.

Results are expressed in pmol/L using a multiplying conversion factor of 44 from ng/mL to pmol/L. Values are given as mean ± SD. Data were analysed using SPSS for windows version 22.0.0.2. Agreement between assays and between the serum and EDTA values were assessed using Passing-Bablock regression, Bland–Altman plots and concordance correlation (CCC) analysis.

## Results

### Quality Assessment

All assays were performed in accordance with the manufacturer’s instructions and complied with our standard operating procedures for good laboratory practice. Inter-assay performance was assessed by calculating the mean, SD and CV % of QC material on 6 plates from the same lot over 2 days for Biomedica and TECO*medical* and 3 plates over 2 days for R&D Systems. CVs were <6 % except for R&D Systems at 15.3 pmol/L where a CV of 14 % was observed (Table 1). We also crossed over the QC material and observed that both R&D Systems and TECO*medical* were close to expected target for each other’s QC (except for low level QC TECO*medical*), however, they both underestimated Biomedica QC by 25–43 %. QCs from TECO*medical* and R&D Systems were mainly overestimated when measured with the Biomedica kit. In order to estimate the intra-assay imprecision, we calculated the average CV from duplicates of samples and also run a serum pool four times on two different plates. Results, presented in Table 1, showed that TECO*medical* performed best with CV < 4.5 % and only 1 sample with a CV > 10 %. However, both Biomedica and R&D Systems showed high CVs on serum and EDTA with CVs up to 35 % for R&D Systems and 69 % for Biomedica). Similar results were obtained with the serum pool run in quadruplicate as Biomedica showed a CV of 33 %.

**Table 1:** Intra- and inter-assay data for the measurement of sclerostin using Biomedica, TECO*medical* and R&D Systems kits

Intra-assay	Mean of %CV ± SEM (maximum %CV)		Serum pool
	Serum (*n* = 46)	EDTA (*n* = 27)	% CV plate 1 and 2
Biomedica	8.2 ± 1.6* (68.8 %)	7.6 ± 1.2* (20.3 %)	33 and 9.9 %
TECO*medical*	2.7 ± 0.4 (11.3 %)	2.7 ± 0.5 (8.9 %)	4.5 and 2.8 %
R&D Systems	5.0 ± 1.1 (35 %)	7.3 ± 1.0* (16.7 %)	9.2 and 3.9 %

Inter-assay	Biomedica	TECO*medical*		R&D Systems		
Mean (pmol/L)	87.7	8.2	92.1	9.2	15.3	37.7
SD	2.8	0.4	3.6	0.2	2.2	2.2
CV	3.2	4.4	3.9	2.4	14.3	5.8
QC cross-over in pmol/L (deviation to target %)						
Biomedica QC		23.6 (+187)	144.6 (+57)	18.3 (+98)	23.9 (+56)	44.3 (+17)
TECO*medical* QC	50.2 (−43)			9.2 (−0.3)	14.5 (−5.3)	34.3 (−9.1)
R&D systems QC	65.6 (−25)	6.2 (−25)	88.3 (−4)			

Intra-assay was estimated using the mean ± SEM of the CVs from samples run in duplicates and a serum pool run six times on two different plates. Inter-assay was estimated by repeated measure of QC material on different plates

Statistical significance, * *p* < 0.05 as compared to TECO*medical*

We assessed the linearity (Table 2) of the assays by diluting serum and EDTA samples by two-, four- and eight-fold using the sample diluent provided in the kits. Upon 1:2 and 1:4 dilutions, sclerostin concentrations were 111 & 89 % and 97 & 103 % of the expected concentration for R&D Systems and TECO*medical,* respectively. Upon 1:8 dilution TECO*medical* sclerostin concentration was 107 % of the expected concentration for TECO*medical;* however, as the neat concentration of the samples were already very low, 1:8 dilution lead to irrelevant values when measured using the R&D Systems assay. When using the Biomedica assay, samples were consistently over-recovered upon dilution (146, 147 and 139 % after 1:2; 1:4 and 1:8 dilution).

**Table 2:** Linearity and recovery data for the measurement of serum sclerostin using Biomedica, TECO*medical* and R&D Systems kits

	Linearity (% ± SEM)			Recovery (% ± SEM)
	1:2	1:4	1:8	
Biomedica	146.7 ± 18.3*	147.4 ± 16.9*	139.1 ± 7.0*	100.6 ± 4.1
TECO*medical*	97.2 ± 3.5	103.2 ± 3.8	107.1 ± 7.1	97.4 ± 4.7
R&D Systems	110.6 ± 18.6	88.5 ± 14.2	685.4 ± 79.1***	97.6 ± 3.0

Linearity was assessed by diluting samples up to eight-fold. Recovery was assessed by spiking samples with known concentration of QC material

Statistical significance, * *p* < 0.05 Biomedica versus TECO*medical* and R&D Systems, *** *p* < 0.001 R&D Systems versus TECO*medical* and Biomedica

Spiked recovery (%) was determined by adding a known quantity of purified sclerostin (from QC material with each assay) to samples containing a range of endogenous sclerostin. Results (Table 2) were very similar between the kits and close to 100 % with Biomedica: 100.6 ± 4.1 %; TECO*medical*: 97.4 ± 4.7 % and R&D Systems: 97.6 ± 3.0 %.

### Sclerostin Measurements

Samples were analysed at the same time using all three kits so differences could not be attributed to differences in sample handling such as freeze/thaw cycles. For each provider, assays were performed using the same lot number and the samples had only been through one freeze–thaw cycle. Recommended maximum freeze–thaw cycles were 4 for Biomedica and 3 for TECO*medical* (no data available for R&D Systems). Table 3 shows mean ± SEM of sclerostin as well as minimum and maximum values obtained with the different assays and depending on collection tube.

**Table 3:** Sclerostin concentrations measured with R&D Systems, TECO*medical* and Biomedica kits in pmol/L and presented as mean ± SEM along with the minimum and maximum values (and the SD)

	Serum (SOST) pmol/L *n* = 46		EDTA (SOST) pmol/L *n* = 27	
	Mean ± SEM	Min–max (SD)	Mean ± SEM	Min–max (SD)
Biomedica	35.5 ± 1.1**^,††^	22.3–58.9 (7.3)	39.4 ± 2.0	22.9–71.1 (10.3)
TECO*medical*	21.8 ± 0.7	11.4–32.6 (4.8)	27.2 ± 1.3	13.8–49.1 (6.9)
R&D Systems	7.6 ± 0.4**	2.7–13.2 (2.4)	30.9 ± 1.5	15.4–53.0 (7.8)

** *p* < 0.001 versus TECO*medical;* ^††^ *p* < 0.001 versus R&D Systems

**Fig. 1:**
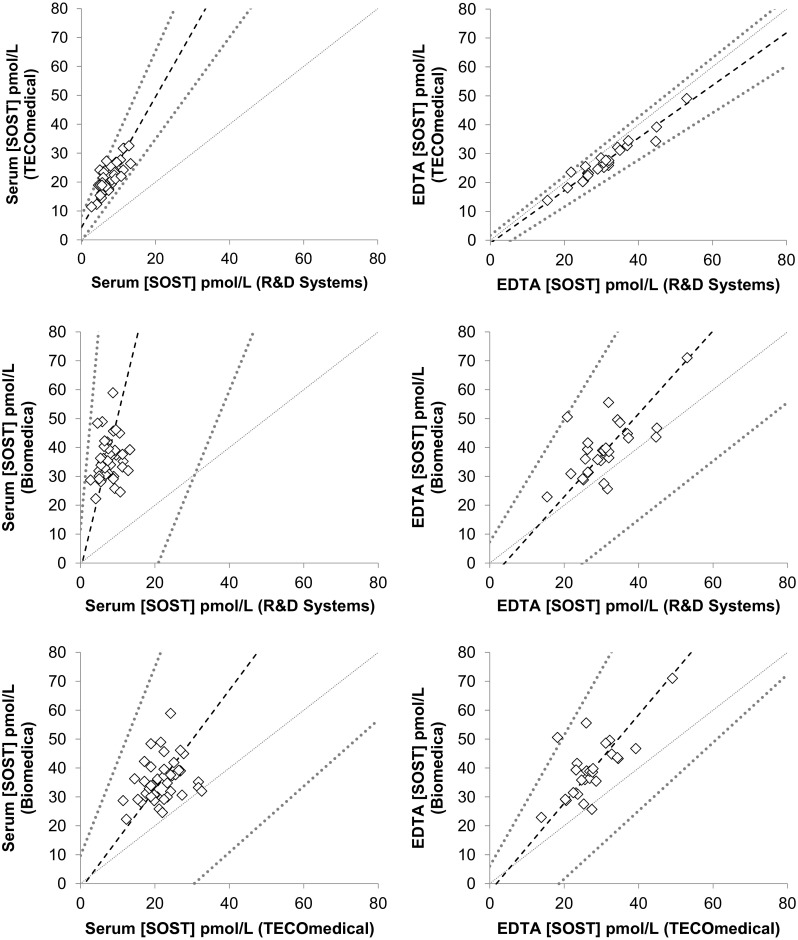
Passing-Bablock regression analysis for serum (*left panel*) and EDTA (*right panel*) samples comparing the three different ELISA kits for circulating sclerostin measurements. *Dash line* represents the fitted regression line; *dark grey dotted lines* represent upper and lower 95 % confidence and *light grey dotted line* represent the identity line

**Fig. 2:**
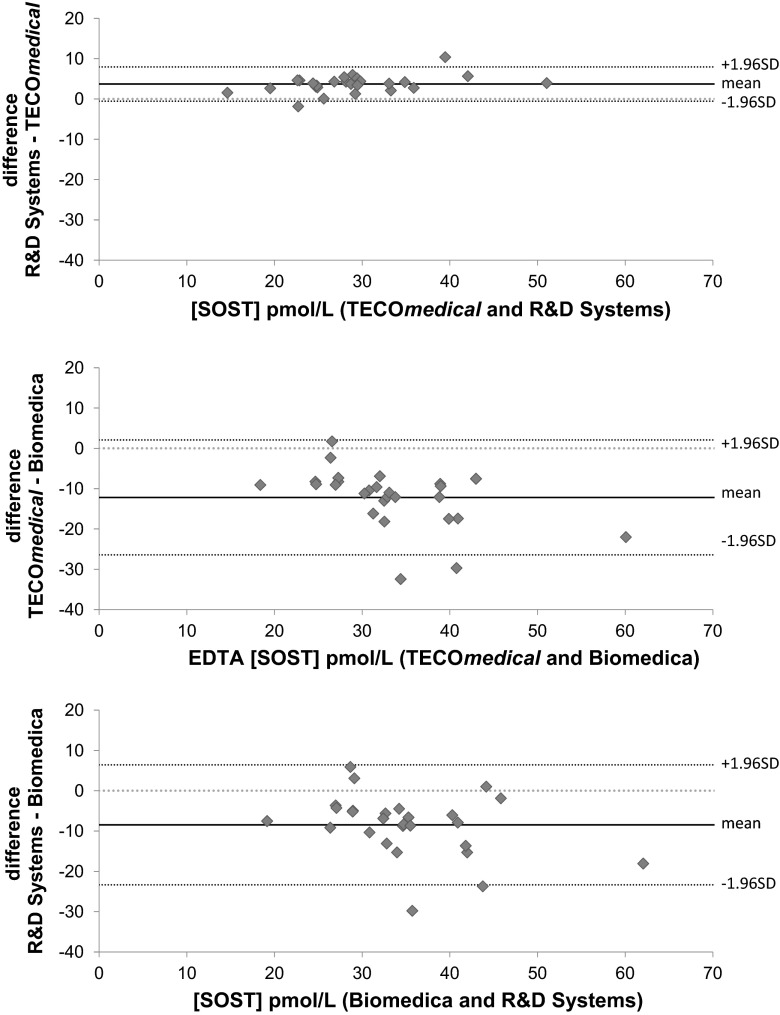
Bland–Altman plots for sclerostin concentrations in EDTA plasma comparing the three different ELISA kits. R&D systems showed little bias when compared to TECO*medical* while both R&D Systems and TECO*medical* assays showed a negative bias (and wide CI) compared to Biomedica; bias present mainly at the highest concentrations of sclerostin

We obtained significantly different values for sclerostin concentrations measured in EDTA plasma samples by each kit. The Biomedica assay detected the significantly highest results up to a concentration of 32 pmol/L and on average, 29.5 % (*p* < 0.001) and 19.8 % (*p* < 0.001) higher than TECO*medical* and R&D Systems assays, respectively. EDTA sclerostin measured by TECO*medical* and R&D Systems were not significantly different. Passing-Bablock regression and concordance correlation analyses (Fig. 1 right panel; Table 4) showed a linear relationship with systematic and proportional differences between the assays when using the EDTA samples. Although R&D Systems gave values 13.8 % lower than TECO*medical*, we observed a good correlation between the two assays when using EDTA samples with Pearson coefficient of 0.96 but a poor agreement between the two methods with CCC of 0.85 (95 % CI 0.738–0.912) and a bias correction of 0.88. Bland–Altman plot showed that the bias was small and similar across the range of concentrations. Biomedica results did not correlate with the other kits and concordance correlations were very poor (CCC of 0.46 and 0.33 versus R&D Systems and TECO*medical,* respectively, with 95 % CI 0.223–0.627 and 0.153–0.486 and Pearson coefficient of 0.68 and 0.71; correction bias were 0.67 and 0.47). Bland–Altman plots (Fig. 2) also showed the bias increased with higher the concentration of sclerostin.

**Table 4:** Passing-Bablock and Lin’s concordance correlation analyses comparing sclerostin ELISA kits on EDTA and serum samples

EDTA	Passing-Bablock regression analysis					Concordance correlation analysis			
	Intercept	95 % CI	Slope	95 % CI	Cusum test	CCC	95 % CI	*r*	Cb
R&D Systems versus TECO*medical*	−1.0	−4.68–1.73	0.9	0.81–1.0	No	0.846	0.738–0.912	0.964	0.878
Biomedica versus R&D Systems	−5.7	−25.5–7.3	1.4	1.0–2.1	No	0.455	0.223–0.627	0.681	0.667
Biomedica versus TECO*medical*	−2.8	−22.0–6.0	1.5	1.2–2.7	No	0.330	0.153–0.486	0.710	0.464

SERUM	Passing-Bablock regression analysis					Concordance correlation analysis			
	Intercept	95 % CI	Slope	95 % CI	Cusum test	CCC	95 % CI	*r*	Cb
R&D Systems versus TECO*medical*	4.3	−0.3 to 8.4	2.6	1.7–2.8	Yes	0.077	0.041 to 0.113	0.780	0.099
Biomedica versus R&D Systems	−2.3	−65.4 to 11.7	5.3	3.2–14.2	No	0.007	−0.006 to 0.02	0.175	0.041
Biomedica versus TECO*medical*	−1.9	−35.0 to 9.7	1.7	1.1–3.3	No	0.076	−0.008 to 0.158	0.288	0.263

*CI* confidence interval; *CCC* concordance correlation coefficient; *r*: Pearson correlation coefficient; *Cb* bias correction. EDTA results showed good correlation between the kits (*r* > 0.68). However, results obtained with the Biomedica kit using serum samples did not correlate with either TECO*medical* (*r* = 0.29) or R&D Systems (*r* = 0.18) and showed poor concordance (CCC < 0.08)

**Fig. 3:**
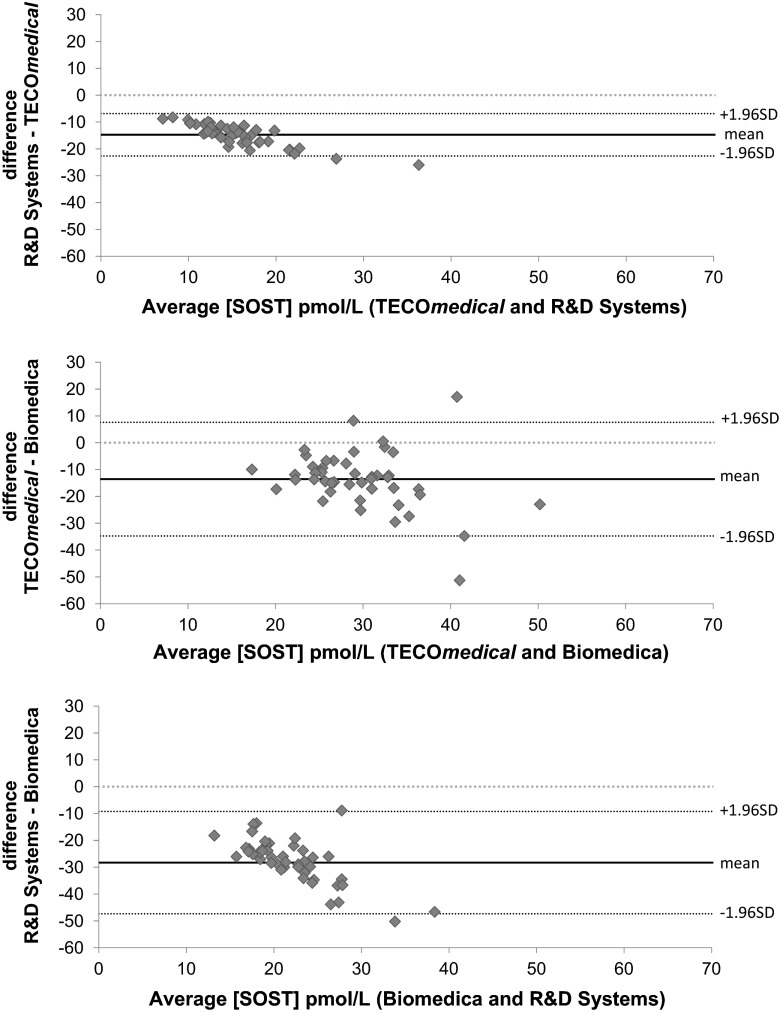
Bland–Altman plots for sclerostin concentrations in serum comparing the three different ELISA kits. R&D systems showed a negative bias when compared to TECO*medical* as well as Biomedica that proportionally increased with increasing concentrations of sclerostin. TECO*medical* showed a negative bias compared to Biomedica which affected mainly the highest concentrations of sclerostin

Larger discrepancies were observed with serum samples. Biomedica gave an average of 37.1 and 77.9 % higher concentrations versus TECO*medical* and R&D systems, respectively (Fig. 1 left panel; Table 3); discrepancies being up to 50 pmol/L (*p* < 0.001). Biomedica results showed very poor correlation with the two other kits with extremely poor concordance between the results (CCC < 0.08 and Pearson correlation coefficient < 0.29). TECO*medical* and R&D Systems correlated with *r* = 0.78, however, R&D systems gave lower results (65 % on average) and extremely poor value agreement (CCC of 0.08 95 %CI 0.041–0.113) and Cusum test for linearity indicates significant deviation from linearity (*p* < 0.05). Bland–Altman plots (Fig. 3) showed a negative bias that increased proportionally as the concentrations increases for both R&D Systems versus TECO*medical* and R&D Systems and Biomedica. When comparing TECOmedical to Biomedica, Bland–Altman plot showed that the bias was mainly present for high concentrations of sclerostin.

### Matrix Effect

**Fig. 4:**
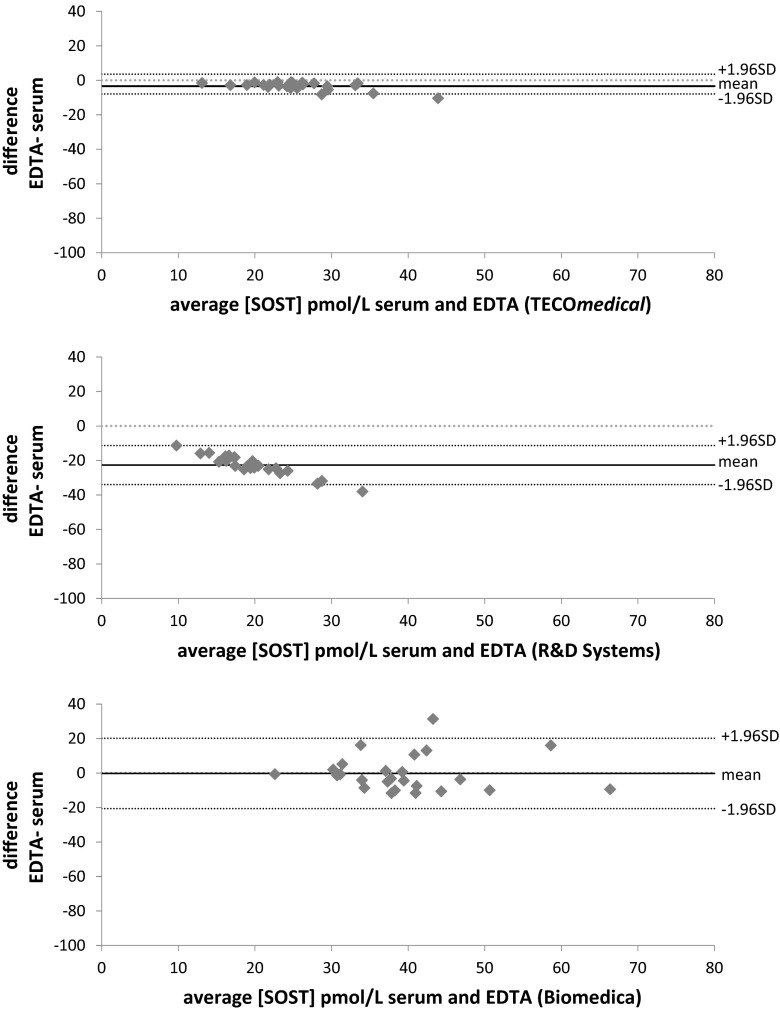
Bland–Altman plots comparing sclerostin concentration in serum versus EDTA plasma using the three different ELISA kits. TECO*medical* showed very little bias between serum and EDTA samples. There was a systematic and proportional negative bias with the R&D Systems assay (from −11 to −38 pmol/L). The bias was present mainly for the high concentrations of sclerostin using the Biomedica assays

Serum sclerostin concentrations (Table 3) were significantly lower than EDTA for TECO*medical* (serum: 21.8 ± 4.8 pmol/L versus EDTA 27.2 ± 6.9 pmol/L *p* < 0.03) and for R&D Systems (serum: 7.6 ± 2.4 pmol/L versus EDTA 19.0 ± 4.7 pmol/L *p* < 0.01) which also displayed a very low CCC of 0.04 although the correlation coefficient was 0.85 in the Passing-Bablock analysis (Table 4; Fig. 4). Passing-Bablock analysis also showed good correlation but poor correspondence of serum and EDTA results for TECO*medical* (CCC of 0.78 95 %CI 0.63–0.87 and Pearson correlation coefficient of 0.9). When using the Biomedica kit, serum and EDTA showed poor correlation (0.20) with differences between −26.6 and 113.9 % and a no agreement with a very low CCC of 0.2. Bland–Altman plots showed that there was virtually no bias between EDTA and Serum measurements when using the TECO*medical* kit, there was a negative bias that proportionally increased with increasing concentrations of sclerostin when using R&D Systems kits and the bias was present mainly at the highest concentrations for Biomedica, however, the CI was larger than the two other kits.

## Discussion

We analysed serum and matching EDTA plasma samples from healthy young men and women aged 18–26 years using commercially available kits from Biomedica, TECO*medica*l and R&D Systems. In general the kits performed according to the manufacturer’s inserts for inter-assay characteristics (performed on QC material) as well as spiked recovery. The Biomedica assay showed a poor linearity on dilution of samples with diluted recovery of 145 %. It has been suggested that heparin as an anticoagulant could interfere with the binding of sclerostin to proteins such as LRP5/6 and the antibodies used in some assays [24]; we therefore used only EDTA plasma and serum samples in this evaluation. Overall, we found that measurements on EDTA samples were more comparable between assays (lower differences in values and better correlations between kits) than on serum samples. This difference could be due to the separation technique. During coagulation in serum samples, clot formation removes proteins such as fibrinogen from the blood sample potentially trapping part of the sclerostin. During the clotting process, platelets get activated inducing the release of various metabolites, which can alter analyte levels relative to plasma [25–27].

The Biomedica assay gave systematically the highest results on samples or QC material from the two other kits. R&D Systems, on the other hand, produced very low values when using serum samples. Cross-over measurement of QC material showed that both R&D Systems and *TECOmedical* were relatively accurate and close to the expected target for each other’s QC, however, both underestimated Biomedica QC. The discrepancies, we observed between the sclerostin concentrations (as for the reference ranges) suggest that the three assays are measuring different forms of the protein and/or the specificity of the antibody used is different. As previously reported, Biomedica and TECO*medical* assays can be affected by interfering substances [28] which may partly account for the poor linearity and the high CVs observed when using the Biomedica kit.

We found poor correlation (0.3) and no agreement of values between TECO*medical* and Biomedica assays using serum samples. These results are conflicting with a similar study from Costa et al. [28] who showed a correlation of 0.9 with systematic and proportional differences between the two methods. This difference could be attributed to the fact that Costa et al. used the Sclerostin TECO^®^ kit which is the previous sclerostin assay developed by TECO*medical* while we used the new version of the assay marked as Human Sclerostin EIA High Sensitivity. This is a different kit, with different antibodies and a different manufacturer’s protocol, which could very well account for the differences in the raw sclerostin values obtained. Also we used samples from pre-menopausal (18–26 year-old) healthy participants while previous studies used samples from a mix of younger and older patients and/or suffering from bone-affecting disorders. Concentrations of sclerostin measured using the Biomedica ELISA, however, were very similar being >30 pmol/L on average in both EDTA plasma and serum; differences quoted are potentially attributable to variation between kit lot numbers. The concentrations of sclerostin we measured using the TECO*medical* assay are ~30 % lower than previously published data in serum and EDTA [24, 28] (but [30] in serum).

This is the first study comparing different kits that included the ELISA assay from R&D Systems. The results indicate comparable raw results and good correlation with TECO*medical* when using EDTA plasma samples. Serum values are lower with the results reflecting those obtained with the Meso Scale Discovery platform [30] of 0.8–3 pmol/L, suggesting that the R&D System kit could be detecting only intact sclerostin. More research is required to answer this question, but variation between lot numbers may be the cause of some of the differences observed.

Given the differences between assays, the results and reference ranges will be assay-specific and specific to sample type. However, comparing mean sclerostin concentrations obtained with mean values quoted by manufacturers for healthy donors, values for TECO*medical* were within the expected reference range. Only 2 and 4 % of samples, respectively, for serum and EDTA were above the reference range for R&D Systems. However, 20 % of serum samples were above the quoted reference range when using the Biomedica assay. No reference range was given for EDTA plasma samples by this manufacturer, however, in their matrix comparison 8 EDTA samples were analysed giving a mean at 18.2 pmol/L leading us to believe a similar gap would be expected between our values and the reference range. This result is in accordance with Moysés et al. [29] who found an extra 25 % of hemodialysed patients were above the reference range when using Biomedica versus TECO*medical.* These differences could have important consequences for patients that are falsely classified as over the range or inversely as presenting normal levels of sclerostin as we cannot to date determine what each assay is actually measuring.

Following its discovery in 2001, interest in sclerostin has expanded in recent years with over 100 publications in 2014. As the knowledge about structure and function of sclerostin is progressively unveiled, sclerostin is being suggested as a “predictor” and “biomarker” for diseases such as chronic kidney disease (CKD) [31], aortic valve calcification [32], osteoporotic fracture [33] or spinal cord injury induced osteoporosis [34]. In CDK in particular, evidence points to a central role of sclerostin in the kidney–bone–vascular axis. As the disease progresses, patients with chronic kidney disease also suffer from vascular calcification and osteodystrophy (CDK-MBD) leading to higher morbidity and mortality. Although the exact pathway is yet to be elucidated and results can be inconsistent, two hypotheses have emerged. Bone-originating sclerostin may have an indirect counter-regulatory action on the vascular calcification via the regulation of the production of other hormones and/or sclerostin may be produced locally when the environment becomes calcifying (for reviews see [23, 35, 36]). Circulating sclerostin concentrations have been reported to vary by sex, age, season and severity of diseases and treatment. The variability in measurement adds to the complexity in drawing conclusions on the role of sclerostin.

As focus grows on sclerostin, authors are rightly being cautious, as we are in this publication, highlighting the need for a consensus and standardization, of the assays to measure sclerostin before sclerostin assays can be used as routine diagnostic tools for metabolic bone diseases. It may be necessary to consider the use of external reference materials for quality control and quality assurance of these assays.
